# The mechanisms associated with the suppression of *Vibrio parahaemolyticus* cells in green-lipped mussels (*Perna canaliculus*)

**DOI:** 10.1093/ismeco/ycag017

**Published:** 2026-01-26

**Authors:** Sinisa Vidovic, Mauro Truglio, Déanna F Shea, Martin J Middleditch, Roland Taylor, Lindy Guo, Xiao Xiao Lin, Sharon Ford, Belinda Timms, Mary A Sewell, Tim D Harwood, Graham C Fletcher

**Affiliations:** The New Zealand Institute for Plant and Food Research Limited, Auckland 1025, New Zealand; New Zealand Food Safety Science and Research Centre, Massey University Manawatu, Palmerston North 4474, New Zealand; Microbiology and Virology Unit, San Gallicano Dermatological Institute, IRCCS, Istituti Fisioterapici Ospitalieri (IFO), Rome 00144, Italy; School of Biological Sciences, University of Auckland, Auckland 1142, New Zealand; School of Biological Sciences, University of Auckland, Auckland 1142, New Zealand; The New Zealand Institute for Plant and Food Research Limited, Auckland 1025, New Zealand; The New Zealand Institute for Plant and Food Research Limited, Auckland 1025, New Zealand; Massey Genome Service, Massey University, Palmerstone North 4442, New Zealand; The New Zealand Institute for Plant and Food Research Limited, Nelson 7010, New Zealand; The New Zealand Institute for Plant and Food Research Limited, Nelson 7010, New Zealand; School of Biological Sciences, University of Auckland, Auckland 1142, New Zealand; New Zealand Food Safety Science and Research Centre, Massey University Manawatu, Palmerston North 4474, New Zealand; Cawthron Institute, Nelson 7042, New Zealand; The New Zealand Institute for Plant and Food Research Limited, Auckland 1025, New Zealand

**Keywords:** *Vibrio parahaemolyticus*, vibriosis, marine microbiota, biofilms, mussels, 16S rRNA, proteomics, biocontrol

## Abstract

The abundance of *Vibrio parahaemolyticus* in contaminated seafood correlates to an infectious dose, which is critical in the occurrence of vibriosis in humans. As *V. parahaemolyticus* does not infect healthy mussels, its interaction with microbiota of mussels can be a key determining factor in infectious dose and the causation of vibriosis in humans. Comparing 216 microbiomes of seawater, biofilms, and mussels over 1 year, we found that the composition of mussel microbiota is different compared to microbiotas of seawater and marine biofilms. Using an *in situ* approach, our results show that mussels with significantly low *V. parahaemolyticus* abundance possess a distinct microbiota in comparison with that of other mussels, biofilms, and seawater. This microbiota is characterized by species of known vibriocidal status (e.g. *Pseudomonas* spp.) and species of unknown vibriocidal status (e.g. *Campylobacterota*, *Bacteroides massiliensis*, *Lancefieldella, Erysipelotrichales*, *Faecalibacterium*, and *Catenibacterium*). Examining proteomes of mussels, we discovered two proteins, the LIM domain-containing protein (**L**in-11, **I**sl-1, and **M**ec-3) and immunoglobulin-like domain protein, constitutively induced only in mussels with low *V. parahaemolyticus* abundance regardless of mussel age or time of harvest. The LIM domain-containing protein showed significant interactions with a group of proteins involved in haemocyte differentiation and endosome biogenesis, key immunological processes. Our results suggest that the low abundance of *V. parahaemolyticus* in mussels likely results from interactions with the resident mussel microbiota and immunological responses of the host.

## Introduction

*Vibrio parahaemolyticus* is the leading cause of seafood-associated gastroenteritis worldwide [1]. A significant change in the epidemiology of *V. parahaemolyticus* occurred during the late 1990s when an increased number of patients were infected with a distinct O3:K6 strain [2]. This newly emerged strain quickly spread to Asia, North America, Europe, and Africa, further causing human infections [3–7]. In 2012, another *V. parahaemolyticus* strain, serotyped as O4:K12 or O4:Kut (sequence type [ST] 36), appeared outside of its endemic area (the Pacific Northwest) [8]. Similar to the first pandemic strain (O3:K6), the geographic expansion of *V. parahaemolyticus* ST36 led to numerous outbreaks [9–12], indicating its great virulence potential [13].

The global change in the epidemiology of *V. parahaemolyticus* cannot be solely explained by the emergence of strains with pandemic potential [14]. The increase in vibriosis associated with *V. parahaemolyticus* is also associated with climate change [15–18]. Vezzulli and colleagues [19], using a combination of archived formalin-preserved plankton samples collected over the past half-century (1958–2011) and generalized additive models, demonstrated that long-term increases in the abundance of *Vibrio* spp. are caused by the increasing sea surface temperature of the North Atlantic (1.5°C increase over 54 years). This finding correlated with the unprecedented occurrence of *V. parahaemolyticus* infections in human populations of Northern Europe and the Atlantic coast of the USA [20].

Interestingly, in New Zealand, the first two outbreaks of *V. parahaemolyticus* took place in winter (June and July of 2019 and 2020) [21], when the monthly average seawater temperature was low, ranging between 14.5°C and 16.1°C [22]. The pandemic ST36 strain was implicated in the first outbreak but was soon replaced by a non-pandemic ST50 strain, which caused three consecutive outbreaks (2020–22). These events highlight further complexity in the vibriosis of *V. parahaemolyticus* and indicate that other factors also play a role. Green-lipped mussels (*Perna canaliculus*), an endemic and important aquaculture species in New Zealand [23], was implicated in these outbreaks. Like other bivalve molluscs, green-lipped mussels (hereinafter referred to as mussels) are filter feeders, which accumulate large numbers of bacteria in their tissue, further creating a specific and highly dynamic habitat for *V. parahaemolyticus*.

To have a better understanding of other factors that could lead to the prevention or reduction of infections, it is necessary to take a holistic view and consider interactions between *V. parahaemolyticus*, the environment, marine microbiota, and its transient [24] or permanent host [25], bivalve molluscs. Therefore, this study focuses on three marine niches associated with *V. parahaemolyticus*: seawater, marine biofilms, and mussels. While seawater is primarily associated with the planktonic lifestyle of this organism, marine biofilms play a critical role in its sessile lifestyle. Finally, mussels, as the filter feeders, contain large numbers of *V. parahaemolyticus* and are the most common transmission vector of this human pathogen into the human food chain. The aim of our study was to determine the effect of abiotic and specifically biotic factors and their interactions on the abundance of *V. parahaemolyticus* in its natural habitat/s.

## Material and methods

### Samples collection

**Figure 1:**
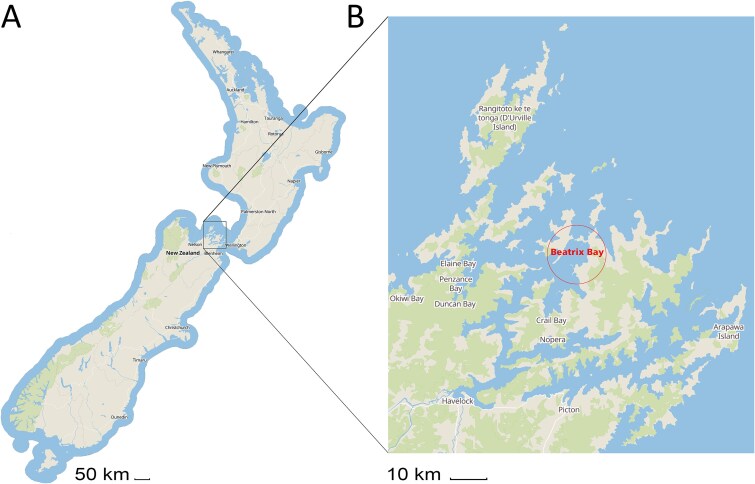
Location of the study site; (A) overall map of New Zealand shows the North Island at the top and the South Island at the bottom; (B) a close-up of the study site, showing the Marlborough Sounds at the northern tip of the South Island indicated Beatrix Bay, an actual study site.

The sampling site was located at Beatrix Bay (41°02′54.6″S 173°59′27.2″E), a region positioned at the northeastern tip of the South Island of New Zealand (Fig. 1A). Beatrix Bay has a circular shape (Fig. 1B), and mean depth of 35 m [26]. Three types of samples, seawater, marine biofilms, and mussels were collected each month over 1 year from January 2022 to January 2023. At each sampling time, the marine biofilms were 1 month old, while the mussels were 1 month older than those from the previous sampling (month). Approximately 2 l of seawater was taken aseptically from a depth of 4 m using a Niskin sample bottle, model 1010 (General Oceanics, Florida, USA). The seawater samples were placed in sterile plastic bottles. To sample marine biofilms, six manually prepared polycarbonate coupons (1 x 10 cm) were attached to a panel and submerged 4 m underwater. One-month-old biofilms were taken and aseptically placed in sterile 50 ml tubes, with a small amount of seawater to prevent biofilm desiccation. Additionally, each month, 78 mussels were collected from a depth of 4 m at a nearby aquaculture farm, located ~20–25 m from the biofilm and seawater sampling site. The mussels were divided into six groups of 13 individuals each. Every month, six samples of seawater, biofilms, and mussels were collected, resulting in a total of 216 samples (72 each type). All samples were stored and transported in coolers with frozen cold packs and processed within 24 h of collection.

### Seawater measurements

A YSI Pro1020 water quality meter (YSI Inc., OH, USA) was used to measure seawater temperature, while an OxyGuard Handy Polaris dissolved oxygen (DO) meter (OxyGuard International A/S, Farum, Denmark) was used to determine DO in seawater. Salinity was measured via a hand-held refractometer (S/Mill-E; ATAGO CO., LTD, Tokyo, Japan) by placing 3 ml of seawater on the refractometer stage (probe depth ~300 mm). The seawater measurements were carried out weekly from January 2022 to January 2023.

### Sample processing and DNA extraction

Seawater samples were filtered using a gridded polytetrafluoroethylene (PTFE) membrane (Sartorius Stedim Biotech GmbH, Göttingen, Germany) with a 0.25-μm pore size. After filtration, each PTFE membrane was aseptically placed in a sterile 50 ml tube, containing 20 ml of sterile molecular grade water (UltraPure^TM^, Life Technologies, Grand Island, NY, USA), and stored at −80°C until further processing. One-month-old marine biofilms were aseptically scraped from the walls of polycarbonate coupons in 10 ml of molecular-grade water and stored at −80°C. The bacterial genomic DNA from seawater and biofilm samples was extracted using a Power Water DNA isolation kit (MO BIO, Carlsbad, CA, USA) according to the manufacturer’s instructions. Regarding the mussel processing, six replicates of 13 mussels were aseptically shucked and tissues of mussels, including their intervalvular water, were homogenized using Power Bead Pro tubes (Tissue Lyser II, Qiagen, Hilden, Germany). Genomic DNA was eluted using an magnetic bead (MB) spin column. To increase the DNA concentration from the seawater and biofilm samples, extracted DNA was concentrated and purified using a DNA Clean & Concentrator kit (Zymo Research, Irvine, CA, USA). All DNA samples used for 16S rRNA sequencing met the purity requirement, with A260/A280 values >1.8.

### Barcoding, amplification, and sequencing

Good quality DNA samples (A_260_/A_280_ > 1.8), and high molecular weight band >10 kb on a 2% agarose gel, were normalized to 10 ng/μl. The 465 bp V3–V4 regions of the 16S rRNA gene were amplified with the following barcoded primers, 341F (5′-AATGATACGGCGACCACCGAGATCTACACxxxxxxxxTATGGTAATTGGCCT-ACGGGAGGCAGCAG-3′) and 806R (5′-CAAGCAGAAGACGGCATACGAG-ATxxxxxxxxAGTCAGTCAGCCGGACTACHVGGGTWTCTAAT-3′). The barcodes were marked with ‘xxxxxxxx’ sign. The polymerase chain reaction (PCR) amplification was carried out in a 20 μl volume, containing 17 μl of AccuPrime™ *Pfx* Super Mix (Invitrogen™), 0.2 mM of each primer, and 50 ng of genomic DNA template. The thermal cycling PCR programme consisted of a DNA predenaturation cycle for 2 min at 95°C, 30 cycles at 95°C for 20 s, 55°C for 15 s, and 72°C for 5 min, followed by a final extension cycle for 20 min at 72°C. A negative control was included in each PCR run. The libraries were purified using SequalPrep™ Normalization Plate Kit (Applied Biosystems™). The full library size of ~630 bp was verified on a LabChip® GX Touch HT instrument using the DNA high sensitivity LabChip® assay. The libraries were pooled by equal molarity and diluted to 2 nM with 10 mM Tris pH 8.5 with 0.1% Tween 20 for sequencing. Approximately, 10 μl of the 2 nM pooled library was denatured to single-stranded DNA by adding 10 μl 0.2 N NaOH (pH > 12.5), followed by mixing and incubating the mixture at room temperature (RT) for 5 min. Illumina PhiX Control v3 was diluted to 2 nM with buffer (10 mM Tris pH 8.5, 0.1% Tween 20) and denatured with 10 μl of 0.2 N NaOH (pH > 12.5), followed by incubation of the mixture at RT for 5 min. The denatured pooled library was diluted to 5 pM, and the denatured PhiX was diluted to 12.5 pM with ice cold HT-1 from MiSeq reagent Kit v2, 500 Cycles. Finally, 800 μl of the pool library and 200 μl of PhiX were combined to give a calculated spike of 20% PhiX. Samples were mixed and 600 μl was loaded into a thawed Illumina MiSeq V2 cartridge for sequencing on the Illumina MiSeq platform.

### Quantitative real-time PCR and most probable number

The quantitative real-time PCR (qPCR) [27] combined with the most probable number (MPN) method [28] was employed to detect and quantify *V. parahaemolyticus*. Briefly, three aliquots of 30 g of homogenized mussel sample were each added to 270 ml of phosphate-buffered saline (PBS), and a further three aliquots of 1 ml were added to 9 ml of PBS, followed by six decimal dilutions giving a detection limit of 0.013 MPN/g. The biofilm was scraped into molecular grade water and vortexed for 1 min. Three 1-ml aliquots of seawater were added to 9 ml alkaline peptone water (APW) followed by six decimal dilutions giving a detection limit of 1.07 MPN/cm^2^. Each PTFE filter from a 2 l seawater sample was vortexed (1 min) in 20 ml of sterile water and three aliquots of 1 ml added to 9 ml of APW followed by six decimal dilutions giving a detection limit of 1.19 MPN/l seawater. Each dilution was incubated at 37°C overnight. After incubation, 1 ml of each turbid solution was boiled at 100°C for 10 min to extract DNA. The qPCR reactions were carried out in a 20-μl reaction mixture containing 250 nM each of the *tdh* and *trh* primers, 150 nM of the *tlh* primers (IDT, NZ), and 75 nM probes for *tlh*, *tdh*, and *trh* (IDT, NZ). Amplification of DNA was carried out using 2 μl of DNA template in SsoFast EvaGreen Supermix (Bio-Rad Laboratories, Hercules, CA) on the 7500 Fast Real-Time PCR System (Applied Biosystems, CA, USA).

### Protein recovery and quantification

Approximately, 500 μl of homogenized mussel tissue was reconstituted in 5 ml of molecular grade water and centrifuged (Eppendorf 5415R) for 20 min at 2000 *g* (4°C) followed by filtration of the supernatant through a 0.45-μm pore size (Merck). Aliquots containing 50 μg of protein, as quantified by the Bradford assay, from each sample were probe sonicated in 100 μl urea/thiourea buffer (7 M urea, 2 M thiourea, 50 mM ammonium bicarbonate) using a Qsonica Q125 ultrasonic processor (Qsonica LLC, Newtown, CT, USA) for 4 x 30 s at 30% amplitude. Disulphide bonds were reduced by incubation at 56°C in a Discover chilled microwave (CEM Corp, Matthews, SC, USA) using 50 W power for 15 min with the addition of 0.75 μl of 1 M dithiothreitol (DTT) to the samples to 5 mM final concentration. Immediately following reduction, cysteines were alkylated by the addition of 1.5 μl of 1 M iodoacetamide (IAM) to 14 mM final concentration, and the samples were incubated in the dark at RT for 25 min, followed by the addition of 1.5 μl of 1 M cysteine to quench residual IAM. Total protein for each sample was quantified by the Bradford assay as per the manufacturer’s instructions. Aliquots containing 50 μg of protein were diluted 10-fold in 50 mM ammonium bicarbonate to permit trypsin digestion with 1.0 μg sequencing grade porcine trypsin (Promega, WI, USA) at 45°C for 1 h. Digested samples were acidified to pH 3 via the addition of 50% formic acid, centrifuged for 2 min at 16 000 *g*, and desalted using 10 mg OASIS HLB SPE cartridges (Waters, MA, USA) as per the manufacturer’s instructions. Peptides were eluted with 300 μl of 50% acetonitrile in 0.1% formic acid, and the eluates were concentrated to a final volume of ~20 μl in a vacuum centrifuge (Thermo Savant, NY, USA). Aliquots of the final extracts were diluted with 0.1% formic acid for liquid chromatography–tandem mass spectrometry analysis.

### Liquid chromatography–tandem mass spectrometry

Sample extracts were analysed neat with 0.1% formic acid, and 2 μl was injected (as appropriate based on signal intensity) onto a 0.3 x 10 mm trap column packed with 3 μm ProteCol C18 media (Trajan Scientific). The samples were desalted for 3 min at 15 μl/min before being separated on a 0.075 x 200 mm PicoFrit column (New Objective) packed in-house with 3u Reprosil C18-AQ media. The following gradient was applied at 300 nl/min using a NanoLC 400 UPLC system (Eksigent): 0 min 1%B; 0.1 min 5%B; 16 min 40%B; 17 min 98%B; 19.5 min 98%B; 20 min 1%B; 30 min 1%B, where A was 0.1% formic acid in water, and B was 0.1% formic acid in acetonitrile. The PicoFrit spray was directed into a TripleTOF 6600 Quadrupole-Time-of-Flight mass spectrometer (SCIEX) for information-dependent analysis (IDA), comprising a time-of-flight mass spectrometry (TOF-MS) scan from 300 to 2000 m/z for 150 ms, followed by 30-ms MS/MS scans on the 30 most abundant species (m/z 80–1600) for a total cycle time of ~1.1 s. The mass spectrometer and ultra-performance liquid chromatography (UPLC) system were under the control of the Analyst TF 1.8 software package (SCIEX).

### Protein identification and annotation

The resulting proteomics data were searched against an ‘in-house’ *P. canaliculus* database comprising of entries for the proteins of interest (21 732 protein entries in total). The search database was used within Protein Pilot version 5.0 with the Paragon Search Algorithm version 5.0.1.0 (AB SCIEX) to identify proteins with the parameters: sample type, identification; search effort, thorough; cysteine alkylation, IAM, digestion; trypsin, ID focus; biological modifications and amino acid substitutions. The peptide summary exported from Protein Pilot was further processed using a custom R-Script to remove proteins with Unused Scores below 0.6, perform false discovery rate (FDR) analysis (with a global threshold of 1%), eliminate inferior or redundant peptide spectral matches, and to sum the intensities for all unique peptides from each protein. Spectral matches for different charge states of the same peptide sequences were retained. Consensus functional annotations of identified proteins were determined using protein basic local alignment search tool (BLASTP) against the National Center for Biotechnology Information (NCBI) database of mussel from the *Mytilus* species (*Mytilus trossulus*, and *Mytilus californianus*) with the Mascot search engine (Matrix Science Ltd, London, UK) as previously described [29].

### Statistical analyses and bioinformatics

A sample count of *V. parahaemolyticus* represents the averages from six biological and three technical replicates. All analyses were carried out using R language version 4.4.1 [30]. *V. parahaemolyticus* abundance data (MPN/g or ml) were log-transformed (log base e) and then analysed with analysis of variance (ANOVA) to test the data effect. Post hoc pair-wise differences between fitted means were tested using Benjamini-Hochberg method at the 5% significance level. Raw DNA sequencing reads were subjected to quality control and preprocessing using Fastp v0.23.4 [31]. The 16S rRNA analysis pipeline the Qiime2 v2023.8 software package [32] was utilized for the subsequent 16S rRNA analysis. The pipeline incorporates the DADA2 algorithm [33] for sequence quality control, chimaera checking, and amplicon sequence variant (ASV) clustering. For the evaluation of alpha diversity, the Shannon index was employed as calculated by the ‘qiime diversity alpha’ command with default parameters. Statistical significance between groups was assessed using a non-parametric Kruskal–Wallis H-test with multiple test corrections employing the Benjamini-Hochberg procedure. Beta diversity was assessed using the unweighted UniFrac distance metric generated by the ‘qiime diversity beta’ command. To examine the statistical differences in microbial community structure among groups, the permutational multivariate analysis of variance (PERMANOVA) was employed. The analysis was conducted with 999 permutations to robustly estimate *P*-values. Alpha and beta diversity results were visualized using the ‘ggplot’ package in R. The effect size of abiotic factors (temperature, salinity, and dissolved oxygen) on microbial composition was calculated using the following equation *r* = *z*/√*N*, where *z* is the *z*-statistic and *N* is the total sample size. Differential abundance analysis was performed using linear discriminant analysis effect size (LEfSe) [34]. A conservative logarithmic linear discriminant analysis (LDA) score threshold of 3.0 was implemented, along with the default settings for the Kruskal–Wallis test among classes, and a Wilcoxon rank-sum test for pairwise comparisons. Gene ontology (GO) enrichment analysis was carried out to determine the biological processes and Kyoto encyclopedia of genes and genomes (KEGG) pathways. The GO analysis was performed using eggNOG-mapper v2 [35] and eggNOG 5.0 [36]. Protein–protein interaction analyses were carried out using the STRING (version 12.0) algorithm [37] applied to an ‘in-house’ database of *P. canaliculus*.

## Results

### Seawater parameters

The seawater temperature reached a maximum monthly average value of 19.3°C in January and then gradually declined until it reached a minimum value of 11.8°C in August (Supplementary Fig. S1A). After this, the temperature gradually increased again, reaching 18.2°C in January (Supplementary Fig. S1A and B), which is a typical temperature pattern in the Southern Hemisphere. The amount of dissolved oxygen showed a constant fluctuation (Supplementary Fig. S1A), ranging from 8.6 to 11.1 mg/l (Supplementary Fig. S1B). The average values for the salinity ranged from 23.5 to 35.5 ppt (Supplementary Fig. S1B). These values for all months exceeded 30 ppt, except for August (23.5 ppt), where a major flooding event played a role in lowering the average salinity in seawater.

### Occurrence and abundance of *V. parahaemolyticus* in seawater, marine biofilms, and mussels

**Figure 2:**
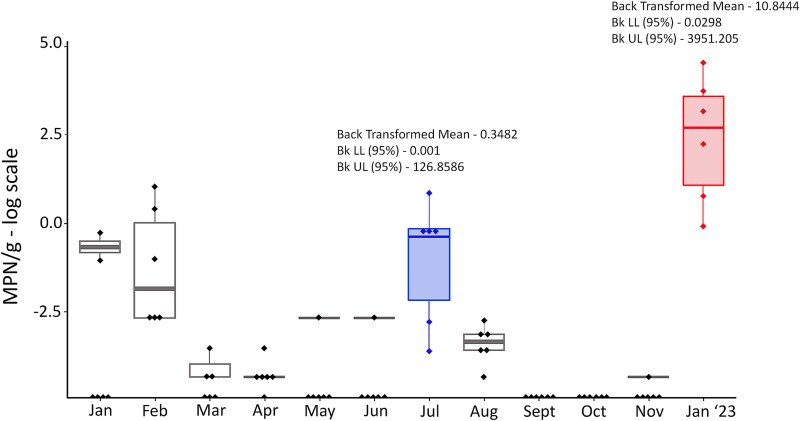
The abundance of *Vibrio parahaemolyticus* in mussels; each month, 78 green-lipped mussels were harvested from the same location and processed in the laboratory; these 78 mussels were divided into six groups; each group represents a pool of 13 mussels; mussels were aseptically shucked, and their tissues were homogenized followed by serial dilutions and incubation at 37°C overnight; DNA was extracted and the quantitative real-time PCR was carried out; results are summarized as boxplots with horizontal lines representing the median; abundance data of *V. parahaemolyticus* most probable number (MPN)/g) were expressed on a natural log scale; the greatest variation in the prevalence of *V. parahaemolyticus* in mussels was observed in the samples harvested in January 2023 (10.844[0.030, 3965.205]^★^) followed by July (0.348[0.001126.857]^★^); ^★^ estimated mean detection with 95% confidence interval; the cold colour blue was used to mark the abundance of *V. parahaemolyticus* from mussel samples harvested in July (winter), and the warm colour red was used to indicate the abundance of *V. parahaemolyticus* in mussel samples from January 2023 (summer).

No *V. parahaemolyticus* was detected in 1-month-old marine biofilms during the study duration (Supplementary Table S1). In seawater, *V. parahaemolyticus* was only detected in one of the six samples during January 2022, February and January 2023 (Supplementary Table S2). In mussels, *V. parahaemolyticus* was detected each month except for September and October (Fig. 2). The lowest mean prevalence of 0.002 MPN/g was observed in November, followed by March (0.009 MPN/g), April, May, and June (0.01 MPN/g) (Supplementary Table S3). The greatest mean prevalence of *V. parahaemolyticus* was detected in January 2023 (28.5 MPN/g) followed by February (0.798 MPN/g), July (0.791 MPN/g), and January 2022 (0.182 MPN/g) (Supplementary Table S3). Most variation was observed in samples harvested in January 2023 with abundance ranging from 0.92 to 93.13 MPN/g (log e scale −0.087 to 4.533), followed by July abundance ranging from 0.06 to 2.3 MPN/g (log e scale −2.81 to 0.83) (Fig. 2).

### Seawater temperature is a major driver of microbial composition change in seawater, marine biofilm, and mussels

**Figure 3:**
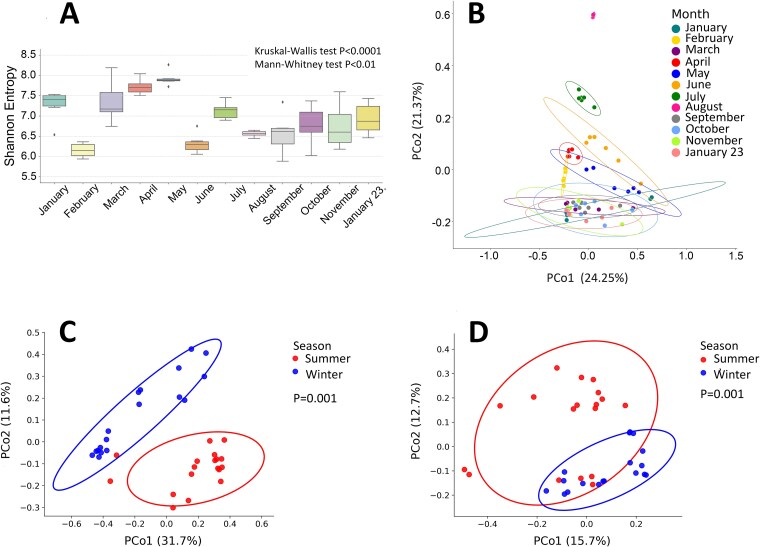
Microbial diversity and community composition vary seasonally across marine habitats; (A) alpha diversity of microbial communities in seawater samples over a 12-month period, measured by Shannon entropy; box plots show median, interquartile range, and outliers; Kruskal–Wallis and Mann–Whitney tests revealed significant differences in microbial richness across months (*P* < .0001); (B) principal coordinates analysis (PCoA) of seawater microbial communities, with samples coloured by month; ellipses represent 95% confidence intervals; significant monthly variations in community composition were observed (*P* = .001, Bray–Curtis dissimilarity analysis); (C) PCoA of biofilm microbial communities and (D) mussel microbial communities, grouped by season; red and blue ellipses represent summer and winter samples, respectively; both biofilm and mussel communities showed significant seasonal differences (*P* = .001), particularly between summer and winter months.

Alpha diversity was calculated to determine the microbial diversity within each sample. By performing the Kruskal–Wallis test, we observed a significant difference (*P* = .007) in microbial richness (i.e. number of different ASVs in a tested microbial community) within mussels. To assess significance for pairwise comparison not covered by the global analysis, we performed Mann–Whitney pairwise post hoc tests. No significant q-value was observed due to FDR corrections. Similarly, a significant difference (*P* < .05) in microbial community richness was observed within biofilm samples using the Kruskal–Wallis test, while no significant value was obtained with the Mann–Whitney test. The seawater samples showed significantly different microbial community richness among months (Fig. 3A), both globally (Kruskal–Wallis test, *P* < .0001) and in pairwise comparisons (Mann–Whitney test *P* < .01 Supplementary Table S4.

To test distances between the microbial communities within each environmental habitat, seawater, biofilm, and mussel, over time, we carried out a Bray–Curtis dissimilarity analysis. The microbial community of seawater showed significant (*P* = .001) differences (Fig. 3B), indicating that the microbial population of seawater could be segregated every month. Similarly, microbial communities of biofilm and mussels each showed significant (*P* = .001) differences mainly between summer (January–February) and winter (June–July–August) months (Fig. 3C and D), suggesting that the seawater temperature plays an important role in shaping the microbial communities of each marine habitat.

Including the monthly and seasonal effects of abiotic factors (seawater temperature, salinity, and dissolved oxygen), seawater temperature showed the significant impact (Supplementary Fig. S2A and B) on the microbial community changes. When the data are analysed monthly, the significant parameters after the Kruskal–Wallis test are associated with the water temperature and salinity (Supplementary Table S5). The post hoc Mann–Whitney U tests with Benjamini-Hochberg FDR correction showed that for water temperature, 54 out of 66 pairwise comparison retain significance, while for salinity, no significance is retained. When the data are analysed as seasonal, the only significant parameters are associated with water temperature and dissolved oxygen (Supplementary Table S5). If we calculate the effect size as *r = z*/√*N*, water temperature shows a large effect, while dissolved oxygen exhibits a medium-low effect (Supplementary Table S6).

While some variations associated with salinity and dissolved oxygen were observed, the additional multiple tests analyses showed that salinity and dissolved oxygen did not exhibit consistent significant patterns (Supplementary Fig. S2C–F). Seawater temperature emerged as the dominant driver of the marine microbial community variation along the primary axis (Supplementary Fig. S3; Supplementary Table S7), suggesting it acts independently rather than as a proxy for other environmental factors like salinity and dissolved oxygen.

### The microbial community of mussels is not influenced by microbiomes of seawater or marine biofilms

**Figure 4:**
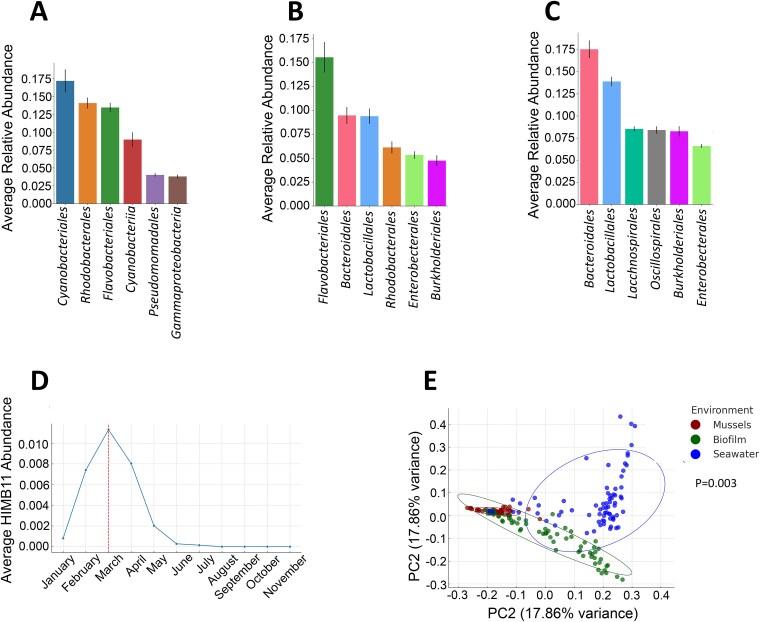
Distinct microbial community compositions characterize seawater, biofilms, and mussels; (A) average relative abundance of the top six bacterial orders in seawater samples over a 12-month period. *Cyanobacteriales* dominated, comprising 17.1% of the total microbial community; (B) average relative abundance of the top six bacterial orders in marine biofilms; *Flavobacteriales* was most prevalent at 15.5% of the total community; (C) average relative abundance of the top six bacterial orders in mussels; *Bacteroidales* dominated at 17.5% of the total microbial community; (D) temporal variation of the genus *HIMB12* (*Rhodobacteraceae* family) in seawater, peaking in late summer/early autumn; analysis performed using QIIME2 volatility analysis with random forests (model accuracy *Pr*^2^ = .84); (E) principal coordinate analysis (PCoA) of microbial communities from mussels, biofilms, and seawater; distinct clustering indicates unique community in each environment (*P* = .003); also, PERMANOVA analysis showed significant differences (Supplementary Table S8) between these three microbial communities, suggesting that they are distinct.

A total of 71 phyla, 151 classes, 442 orders, 808 families, and 1788 (ASVs) were identified in the seawater samples over 12 months. The most abundant order in the seawater was *Cyanobacteriales*, making up 17.1% of the total relative abundance, followed by *Rhodobacterales* (14%), *Flavobacteriales* (13.4%), *Cyanobacteriia* PCC-6307 (9%), *Pseudomonadales* (4%), and *Gammaproteobacteria* SAR86 (3.8%) (Fig. 4A). *Cyanobacteriales* was present in the seawater at each measured time point, indicating it is an integral part of the seawater microbial community. Regarding biofilms, a total of 66 phyla, 147 classes, 422 orders, 768 families, and 1792 (ASVs) were identified throughout the study. The most abundant order was *Flavobacteriales*, making up 15.5% of the total relative abundance, followed by *Bacteroidales* (9.4%), *Lactobacillales* (9.3%), *Rhodobacterales* (6.1%), *Enterobacterales* (5.3%), and *Burkholderiales* (4.7%) (Fig. 4B). Interestingly, no bacterial genus or family showed any significant (*P* = .05) fluctuation over the 12 months, indicating that the microbial community of marine biofilm is more stable compared to seawater under the environmental conditions presented in this study. During the same period, we identified a total of 63 phyla, 140 classes, 407 orders, 763 families, and 1975 (ASVs) in mussels, establishing mussels as the most diverse habitat for the bacterial community. *Bacteroidales* was the most abundant order (17.5%), followed by *Lactobacillales* (13.8%), *Lachnospirales* (8.5%), *Oscillospirales* (8.4%), *Burkholderiales* (8.2%), and *Enterobacterales* (6.5%) (Fig. 4C). *Bacteroidales* were constantly present in mussels, accounting for between 44% and 56% of the total relative abundance over all four seasons. Volatility analysis identified the most significant (*P* = .0039; *r*^2^ = 0.48) variations for genera *Maribacter*_A, *Endozoicomonas*, and *Ruminococcus*_B, indicating their transient nature in the microbiota of mussels.

To determine the temporal variations in the microbial composition of seawater, we carried out volatility analysis within the QIIME2 framework using a Random Forest machine learning model. This analysis revealed a 10-fold increase in the abundance of the genus *HIMB12* from the *Rhodobacteracea* family during February, March, and April [a period that corelates to the late summer and early autumn months (February–April) in the Southern Hemisphere] (Fig. 4D; model accuracy *Pr*^2^ = .84).

To confirm these findings, the relative abundance of seawater, biofilm, and mussels’ microbiomes were analysed using principal coordinate analysis (PCoA) and PERMANOVA. The PCoA analysis resulted in global *P* = .003, and PERMANOVA analysis resulted in the significant pairwise differences, ‘mussels vs biofilm’, ‘mussels vs seawater’, and ‘biofilm vs seawater’ (Fig. 4E), (Supplementary Table S8), suggesting that these three groups are different.

### Genus *Pseudomonas* shows the most significant association with mussels, which contain a low number of *V. parahaemolyticus*

**Figure 5:**
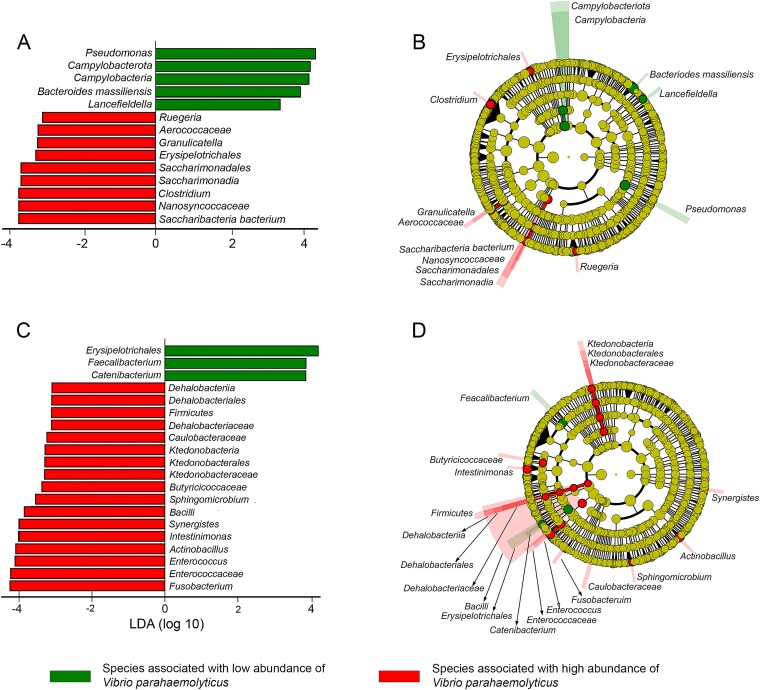
Distinct microbial taxa are associated with mussels containing low numbers of *Vibrio parahaemolyticus*; 16S rRNA data from mussels harvested in summer (January 2022 and 2023) and winter were analysed using LEfSe (linear discriminant analysis effect size), which combines non-parametric statistical testing with linear discriminant analysis to identify microbial taxa significantly associated with different *V. parahaemolyticus* abundance levels (*P* ≤ .05); phylogenetic relationships were visualized using cladograms; (A) LEfSe analysis results showing microbial taxa significantly associated with mussels containing low (green) or high (red) *V. parahaemolyticus* numbers in summer; the length of each bar represents the LDA score (log10 scale); (B) cladogram illustrating the phylogenetic relationships of microbial taxa associated with low (green) or high (red) *V. parahaemolyticus* abundance in mussels during summer; node size corresponds to taxon abundance; (C) LEfSe analysis results for mussels harvested in winter, showing fewer taxa associated with low *V. parahaemolyticus* numbers (green) compared to high numbers (red); (D) cladogram of microbial taxa associated with low (green) or high (red) *V. parahaemolyticus* abundance in mussels during winter, demonstrating less distinct phylogenetic separation compared to summer.

We observed that the numbers of *V. parahaemolyticus* in mussel samples, harvested during the same month, significantly (*P* ≤ .05) differed. The greatest variation in the abundance of *V. parahaemolyticus* in mussels was observed between samples harvested in January 2023 followed by July. To identify microbial taxa that were significantly more associated with mussels containing low numbers of *V. parahaemolyticus*, we performed LEfSe analysis. Mussel samples harvested in January 2023, with significantly (*P* ≤ .05) lower numbers of *V. parahaemolyticus*, showed the strongest association with *Pseudomonas* (*P* = .001; LDA score = 4.33), followed by *Campylobacterota* (*P* = .006; LDA score = 4.2), *Campylobacteria* (*P* = .006; LDA score = 4.19), *Bacteroides massiliensis* (*P* = .04; LDA score = 3.9)*,* and *Lancefieldella* (*P* = .04; LDA score = 3.34) (Fig. 5A). Microbial taxa that were significantly associated with mussels containing low numbers of *V. parahaemolyticus* showed distant phylogenetic relatedness to their counterparts that were significantly associated with mussels containing higher numbers of *V. parahaemolyticus* (Fig. 5B). For mussels harvested during the winter (July), only three genera, *Erysipelotrichales* (*P* = .03; LDA score = 4.2), *Faecalibacterium* (*P* = .03; LDA score = 3.87), and *Catenibacterium* (*P* = .04; LDA score = 3.86), showed significant association with mussels containing a low number of *V. parahaemolyticus* (Fig. 5C). In contrast to the summer, during winter conditions, no distant phylogenetic relatedness was observed among microbial taxa that were significantly associated with mussels containing high and low numbers of *V. parahaemolyticus* (Fig. 5D).

### Proteome analysis

**Figure 6:**
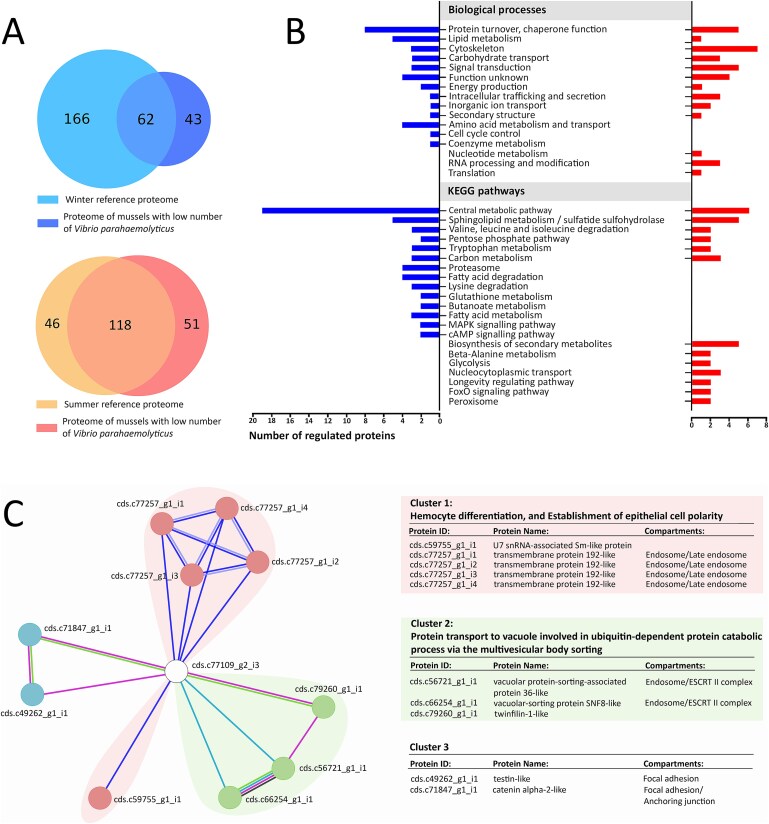
Comparative proteomics analyses of mussels with different *Vibrio parahaemolyticus* abundance; (A) Venn diagrams showing the overall proteome profiles of mussels with significant differences in *V. parahaemolyticus* abundance; proteomes of mussels harvested in winter are presented by blue colour shades, while mussels harvested in summer are presented by red colour shades; note the proteomes of mussels with high *V. parahaemolyticus* abundance served as the reference proteomes; (B) gene ontology enrichment clustering analysis portraying biological processes and KEGG pathways of unique proteins associated with mussels of low *V. parahaemolyticus* abundance; blue bars represent the proteins identified in mussels harvested in winter and red represents proteins identified in mussels harvested in summer; (C) predicted interactome of the LIM domain-containing protein; the interactome is created based on the STRING algorithm and an ‘in-house’ database of 21, 732 green-lipped mussels’ proteins; each protein–protein interaction is annotated with a significant protein–protein interaction enrichment *P* value (*P* < .05), and acceptable false discovery rate (FDR < 0.5); proteins that interact with the LIM domain-containing protein are stratified as three clusters according to their function.

To identify proteins that might be associated with mussels containing low number of *V. parahaemolyticus*, we compared proteomes of these mussels with proteomes of mussels harvested at the same time, which contained higher number of *V. parahaemolyticus* (the reference proteomes). Of the 271 proteins detected for mussels harvested in winter (July), 43 proteins were unique for mussels containing low number of *V. parahaemolyticus* (Fig. 6A). For mussels harvested in summer (January 2023), 51 proteins were unique for mussels with low numbers of *V. parahaemolyticus* (Fig. 6A) (Supplementary Tables S9 and S10).

The most notable biological processes in mussels with low number of *V. parahaemolyticus* harvested in winter were ‘protein turnover and chaperone function’, followed by ‘lipid metabolism’, and ‘amino acid metabolism and transport’ (Fig. 6B). The hallmark of this proteome was the presence of proteasome (Fig. 6B; KEGG pathways) subunits of alpha types 5 and 7 and beta types 3 and 2 (Supplementary Table S10). In mussels with low numbers of *V. parahaemolyticus* harvested in summer, the most distinguishing biological processes were ‘cytoskeleton’, ‘protein turnover and chaperone function’, and ‘signal transduction’ (Fig. 6B). Enzymes, fructose-1,6-bisphosphatase, 3-hydroxyacyl-CoA dehydrogenase, enoyl-CoA hydratase, phosphopentomutase, and catalase (Supplementary Table S9), involved in the biosynthesis of secondary metabolites (Fig. 6B; KEGG pathways), were identified in these mussel samples.

Interestingly, the unique proteins of these two groups of mussels (winter and summer), with low numbers of *V. parahaemolyticus*, were quite different, even when they are involved in the same cellular pathways (Supplementary Tables S9 and S10). Only two proteins, uncharacterized LIM domain-containing protein (cds.c77109_g2_i3 or XP_052064529.1) and leucine-rich repeats and immunoglobulin-like domains protein (cds.c62360_g1_i1 or XP_063430697), were common to both groups of proteomes. We carried out their protein–protein interaction and functional enrichment analyses using an ‘in-house’ produced database of *P. canaliculus*. Uncharacterized LIM domain-containing protein showed significant interactions with proteins involved in two distinct protein–protein clusters: (i) haemocyte differentiation and establishment of epithelial cell polarity and (ii) protein transport to vacuole (Fig. 6C). Significant interactions with two multifunctional proteins, testin-like (cds.c49262_g1_i1) and catenin alpha-2-like isoform X7 (cds.c71847_g1_i1) were also noted (Fig. 6C). No significant protein–protein interactions were identified for immunoglobulin-like domain protein.

## Discussion

There is a significant knowledge gap in understanding the underlying interactions between *V. parahaemolyticus* and important bivalve molluscs aquaculture species, including mussels and oysters. While certain species of the *Vibrio* genus have been associated with the mortality of oysters (*Vibrio aestuarianus*, *Vibrio crassostreae, Vibrio cyclitrophicus, Vibrio splendidus*, *Vibrio tasmaniensis*) [38–40], or mussels (*Vibrio celticus*, *V. splendidus*) [41, 42], there is no literature to date to suggest that *V. parahaemolyticus* causes infection in healthy mussels or oysters. This implies that interactions between *V. parahaemolyticus* and the microbiota of mussels and oysters may be of critical importance in the transmission of this foodborne pathogen to humans. As filter feeders, mussels and oysters, in their natural marine environment, filter large volumes of water [43], thereby creating diverse and rich microbiota [44]. In this host-microbiome assemblage, where *V. parahaemolyticus* does not cause host infection, abundance of this human pathogen may greatly depend on interactions with the host microbiota. Recently, a few studies have investigated the diversity of mussel microbiota [44, 45] or the starvation response of mussels’ gut microbiome [46] but little is known about the interactions of *V. parahaemolyticus* with the microbiota of the healthy bivalve host (mussel). To better understand the factors influencing the abundance of *V. parahaemolyticus*, we took a holistic approach. First, we examined the effects of abiotic factors—seawater temperature, salinity, and dissolved oxygen—on the overall composition of the marine microbial community. Next, we focused on biotic factors, using a longitudinal *in situ* approach to investigate the interactions between *V. parahaemolyticus*, the mussel microbiota, and the mussel hosts themselves.

Regarding abiotic factors, the data from this study suggest that seawater temperature was a major driving force influencing microbial composition change in all three habitats relevant to *V. parahaemolyticus*. Although dissolved oxygen and salinity had some impact, their patterns were inconsistent, became non-significant after multiple-testing correction, and explained less variation than temperature in multivariate analyses. These results align with an earlier study demonstrating the significant effect of seawater temperature change on the composition of microbial communities associated with reef biofilms [47].

In the literature it is documented that the degree of contamination of seafood with *V. parahaemolyticus* plays an important role in the occurrence of potential outbreaks or sporadic infections [48, 49]. The investigation related to the outbreak of *V. parahaemolyticus* associated with the consumption of Alaskan oysters showed that illness could occur with a medium count of 3.5 most probably number of *V. parahaemolyticus* cells per 1 g of oysters, [50]. This level represents a significantly lower infectious dose than previously anticipated for *V. parahaemolyticus* [51], highlighting the complexity associated with human vibriosis.

To determine interactions of *V. parahaemolyticus* with the microbiota of mussels with significantly different abundance of this pathogen, we carried out comparative analyses of their microbiomes. We found that during the summer, *Pseudomonas*, *Campylobacterota*, *B. massiliensis*, and *Lancefieldella* were significantly associated with mussels that contained low numbers of *V. parahaemolyticus*. In another set of mussels harvested in winter, *Erysipelotrichales*, *Faecalibacterium*, and *Catenibacterium* were significantly associated with mussels containing low numbers of *V. parahaemolyticus*. Several studies have shown that *Pseudomonas* spp. can suppress the growth of marine *Vibrio*. Gram *et al*. [52] demonstrated that *Pseudomonas fluorescens* could inhibit the growth of *Vibrio anguillarum,* resulting in a 46% reduction in fish mortality. More recently, Zhang *et al*. [53] demonstrated that *Pseudomonas aeruginosa* can significantly inhibit the growth and reduce biofilms developed by *V. parahaemolyticus* and *V. cyclitrophicus* by secretion of pyoverdine, a potent fluorescent siderophore with high ferric ion (Fe^3+^) affinity.

Although *V. parahaemolyticus* does not cause infection of its bivalve host, Ciacci *et al* [54] reported that this human pathogen induces significant extracellular lysozyme release, resulting in an efficient immune response in mussels. To better understand the interaction of *V. parahaemolyticus* with its host, we carried out comparative proteome analyses between mussels with high and low *V. parahaemolyticus* abundance. Besides proteins involved in the central metabolic pathways, the most notable biological processes in mussels with low number of *V. parahaemolyticus* harvested in winter were ‘post-translational modification, protein turnover, and chaperone functions’. In mussels harvested in summer, ‘cytoskeleton’ followed by ‘post-translational modification, protein turnover, and chaperone function’ and ‘signal transduction’ were most pronounced biological processes. The unique proteins of mussels harvested in winter and summer were quite different from each other, likely reflecting the age of mussels and the biotic and abiotic conditions when harvested. However, two proteins of unknown functions, LIM domain-containing protein and leucine-rich repeats and immunoglobulin-like domain protein, were constitutively induced only in mussels with low *V. parahaemolyticus* abundance regardless of mussel age or environmental conditions. To learn more about the functions of these two proteins, we carried out protein–protein (interactome) analyses. The immunoglobulin-like domain protein showed no significant interactions with any protein in the proteome of *P. canaliculus*. However, the LIM domain-containing protein showed significant interactions with four transmembrane proteins and a U7 snRNA-associated Sm-like protein all involved in haemocyte differentiation and establishment of epithelial cell polarity. Additionally, the LIM domain-containing protein exhibited significant interactions with proteins involved in protein transport to vacuole, and proteins associated with focal adhesion. A great majority of proteins interacting with the LIM domain-containing protein are involved in the biogenesis of endosomes (Fig. 6C). It has been well documented that haemocytes are key players in bivalve immune response [55–57].

Findings from this study shed new light on the complex interactions of *V. parahaemolyticus* with mussels and their microbiota. We demonstrated that mussels with a low abundance of *V. parahaemolyticus* have a distinct microbiota, characterized by microbial species of known vibriocidal status and species of unknown vibriocidal status. The microbial species of unknown vibriocidal status (e.g. *Campylobacterota*, *B. massiliensis*, *Lancefieldella, Erysipelotrichales*, *Faecalibacterium*, and *Catenibacterium*) are potential candidates for future research seeking to discover naturally marine occurring antagonistic organisms to *V. parahaemolyticus*. In addition, we showed that mussels with low *V. parahaemolyticus* abundance constitutively synthesize protein of unknown function, the LIM domain-containing protein. The LIM domain-containing protein showed significant interactions with a group of proteins involved in key immunological responses (haemocyte differentiation and biogenesis of endosomes). It is important to note that this study has limitations, including the use of a single sampling location (Beatrix Bay, New Zealand), which may not represent other geographic regions with different environmental conditions.

## Supplementary Material

Supplementary_Materials_Figures_and_Tables_Legends_ycag017

Supplementary_Tables_ycag017

## Data Availability

The data for this study have been deposited in the European Nucleotide Archive (ENA) at EMBL-EBI under accession number PRJEB80804 (https://www.ebi.ac.uk/ena/browser/view/PRJEB80804). The mass spectrometry proteomics data have been deposited to the ProteomeXchange Consortium via the PRIDE partner repository with the dataset identifier PXD065202: https://checkpoint.url-protection.com/v1/r01/url?o=https://www.ebi.ac.uk/pride/archive/projects/PXD065202&g=ZjViMmZmYmZmMjBmMGZkMA==&h=Mzk3NWEwOTkyZTkwNTNkYWExMmY1YmMwYmI5ZjdjYWQzZTE2NjI1YmYxMDgwNDBkZTk3NTRlYjczMmUzNjAzMw==&p=YzJ1OnBsYW50YW5kZm9vZHJlc2VhcmNoOmM6bzoxYTc3NDIyMmY5YTZhNTZlZWI5ZDQ0MjhhMDFhNTlkNDo3OnQ6VA==.
