# Supplementary material for: The mechanisms associated with the suppression of Vibrio parahaemolyticus cells in green-lipped mussels (Perna canaliculus)
Source: ISME Commun. 2026 Jan 26;6(1):ycag017. doi: 10.1093/ismeco/ycag017 (PMC12904275; doi:10.1093/ismeco/ycag017)
Supplement: Supplementary_Materials_Figures_and_Tables_Legends_ycag017 [file supplementary_materials_figures_and_tables_legends_ycag017.docx]

**Supplementary materials**

**Figure legends**

**Figure S1. Characteristics of seawater, including temperature, dissolved oxygen, and salinity.** (**A**) Weekly measurements of each parameter (temperature, dissolved oxygen, and salinity) from the beginning of January 2022 until the first 10 days of January 2023. (**B**) Colour-coded monthly average values for temperature, dissolved oxygen and salinity correspond to the colour scale bars.

**Figure S2**. **Influence of abiotic factors on the marine microbial community change**. (**A**) Seawater temperature showed the strong significance (P<0.0001) on monthly basis after Kruskal-Wallis test. The post-hoc Mann-Whitney U tests with Benjamini-Hochberg FDR correction showed that for water temperature 54 out of 66 pairwise comparisons retained significance, indicating a consistent significant pattern. (**B**) Seawater temperature exhibited the significant impact (P<0.0001) on seasonal level. (**C**) Salinity showed the significant (P = 0.0112) impact on monthly basis but after FDR correction no significance was retained. (**D**) Salinity showed no significant impact on the seasonal level. (**E**) Dissolved oxygen had no significant effect on monthly basis, whereas during seasons (**F**) dissolved oxygen showed the significant (P = 0.0345) effect.

**Figure S3. Principal Component Analysis (PCA) of seawater temperature, salinity and dissolved oxygen effects on microbial communities**. PC1 explains 49.6% of total variance with water temperature having the highest loading (0.644). PC2 explains 28.6% of variance but water temperature barely contributes (loading -0.073). Seawater temperature is the dominant contributor to the primary axis of variation. The strong opposing loading of dissolved oxygen and salinity on PC2 suggest they have a relationship independent of seawater temperature, but this explains less variation than the temperature-dominant PC1.

**Table legends**

**Table S1.** Abundance of *Vibrio parahaemolyticus* in marine biofilm.

**Table S2**. Abundance of *Vibrio parahaemolyticus* in seawater. Values were expressed as most probable number (MPN) of *V.* *parahaemolyticus* per 1 mL of seawater.

**Table S3.** Abundance of *Vibrio parahaemolyticus* in mussels. Values were expressed as most probable number (MPN) of *V.* *parahaemolyticus* per 1 g of mussel’s tissue.

**Table S4.** Kruskal-Wallis pairwise on monthly bases.

**Table S5.** The monthly and seasonal variations of marine microbiota.

**Table S6.** The effect size of water temperature and dissolved oxygen. z is the z-statistic and N is the total sample size.

**Table S7.** Spearman rank correlations. Based on 68 samples with complete data for all three parameters.

**Table S8.** Pairwise PERMANOVA

**Table S9.** Unique mussel proteins, harvested in summer which contained a significantly lower number of *Vibrio parahaemolyticus*. Proteins were originally identified using an in-house *Perna canaliculus* database (21,732 proteins)^a^. Sequences were searched against an National Center for Biotechnology Information (NCBI) database Basic Local Alignment Search Tool (BLAST)^b^ to obtain publicly available accession identifiers.

**Table S10.** Unique mussel proteins, harvested in winter, which contained a significantly lower number of *Vibrio parahaemolyticus*. Proteins were originally identified using an in-house *Perna canaliculus* database (21,732 proteins)^a^. Sequences were searched against an NCBI database (BLAST)^b^ to obtain publicly available accession identifiers.
