# Supplementary material for: The mechanisms associated with the suppression of Vibrio parahaemolyticus cells in green-lipped mussels (Perna canaliculus)
Source: ISME Commun. 2026 Jan 26;6(1):ycag017. doi: 10.1093/ismeco/ycag017 (PMC12904275; doi:10.1093/ismeco/ycag017)
Supplement: Supplementary_Tables_ycag017 [file supplementary_tables_ycag017.docx]

**Table S1.**

| **Sample** | | **Time** | | | | | | | | | | | |
| --- | --- | --- | --- | --- | --- | --- | --- | --- | --- | --- | --- | --- | --- |
|  |  | Jan-22 | Feb-22 | Mar-22 | Apr-22 | May-22 | Jun-22 | Jul-22 | Aug-22 | Sep-22 | Oct-22 | Nov-22 | Jan-23 |
|  | MB - 1 | ND^1^ | ND | ND | ND | ND | ND | ND | ND | ND | ND | ND | ND |
|  | MB - 2 | ND | ND | ND | ND | ND | ND | ND | ND | ND | ND | ND | ND |
|  | MB - 3 | ND | ND | ND | ND | ND | ND | ND | ND | ND | ND | ND | ND |
|  | MB - 4 | ND | ND | ND | ND | ND | ND | ND | ND | ND | ND | ND | ND |
|  | MB - 5 | ND | ND | ND | ND | ND | ND | ND | ND | ND | ND | ND | ND |
|  | MB - 6 | ND | ND | ND | ND | ND | ND | ND | ND | ND | ND | ND | ND |

^1^ – Not detected.

**Table S2**.

| **Sample** | **Time** | | | | | | | | | | | |
| --- | --- | --- | --- | --- | --- | --- | --- | --- | --- | --- | --- | --- |
|  | Jan-22 | Feb-22 | Mar-22 | Apr-22 | May-22 | Jun-22 | Jul-22 | Aug-22 | Sep-22 | Oct-22 | Nov-22 | Jan-23 |
| MB - 1 | ND | ND | ND | ND | ND | ND | ND | ND | ND | ND | ND | ND |
| MB - 2 | ND | 0.357 | ND | ND | ND | ND | ND | ND | ND | ND | ND | ND |
| MB - 3 | ND | ND | ND | ND | ND | ND | ND | ND | ND | ND | ND | 0.357 |
| MB - 4 | 0.357 | ND | ND | ND | ND | ND | ND | ND | ND | ND | ND | ND |
| MB - 5 | ND | ND | ND | ND | ND | ND | ND | ND | ND | ND | ND | ND |
| MB - 6 | ND | ND | ND | ND | ND | ND | ND | ND | ND | ND | ND | ND |

**Table S3.**

| **Sample** | **Time** | | | | | | | | | | | |
| --- | --- | --- | --- | --- | --- | --- | --- | --- | --- | --- | --- | --- |
|  | Jan-22 | Feb-22 | Mar-22 | Apr-22 | May-22 | Jun-22 | Jul-22 | Aug-22 | Sep-22 | Oct-22 | Nov-22 | Jan-23 |
| MB - 1 | ND | 0.069 | 0.028 | 0.028 | ND | ND | 0.722 | 0.061 | ND | ND | ND | 9.190 |
| MB - 2 | ND | 2.758 | ND | 0.013 | ND | ND | 0.027 | 0.044 | ND | ND | 0.013 | 0.916 |
| MB - 3 | ND | 1.466 | 0.013 | 0.013 | 0.069 | ND | 0.916 | 0.044 | ND | ND | ND | 2.103 |
| MB - 4 | ND | 0.069 | ND | ND | ND | ND | 0.722 | 0.027 | ND | ND | ND | 42.694 |
| MB - 5 | 0.735 | 0.069 | ND | 0.013 | ND | ND | 2.303 | 0.013 | ND | ND | ND | 23.107 |
| MB - 6 | 0.357 | 0.357 | 0.013 | 0.013 | ND | 0.069 | 0.06 | 0.028 | ND | ND | ND | 93.127 |

**Table S4.**

| **Group 1** | **Group 2** | **H** | **p-value** | **q-value** |
| --- | --- | --- | --- | --- |
| 01-January_22 (n=6) | 02-February_22 (n=6) | 8.307692308 | 0.003947752 | 0.010856318 |
| 01-January_22 (n=6) | 03-March_22 (n=6) | 0.102564103 | 0.748774042 | 0.760293642 |
| 01-January_22 (n=6) | 04-April_22 (n=6) | 6.564102564 | 0.01040562 | 0.023681756 |
| 01-January_22 (n=6) | 05-May_22 (n=6) | 8.307692308 | 0.003947752 | 0.010856318 |
| 01-January_22 (n=6) | 06-June_22 (n=6) | 7.41025641 | 0.006485308 | 0.016462705 |
| 01-January_22 (n=6) | 07-July_22 (n=6) | 2.564102564 | 0.109314576 | 0.156842653 |
| 01-January_22 (n=6) | 08-August_22 (n=6) | 5.769230769 | 0.016309172 | 0.035880178 |
| 01-January_22 (n=6) | 09-September_22 (n=6) | 3.692307692 | 0.054663936 | 0.094942625 |
| 01-January_22 (n=6) | 10-October_22 (n=6) | 3.102564103 | 0.078169086 | 0.128978992 |
| 01-January_22 (n=6) | 11-November_22 (n=6) | 2.564102564 | 0.109314576 | 0.156842653 |
| 01-January_22 (n=6) | 13-January_23 (n=6) | 2.076923077 | 0.149541355 | 0.186221309 |
| 02-February_22 (n=6) | 03-March_22 (n=6) | 8.307692308 | 0.003947752 | 0.010856318 |
| 02-February_22 (n=6) | 04-April_22 (n=6) | 8.307692308 | 0.003947752 | 0.010856318 |
| 02-February_22 (n=6) | 05-May_22 (n=6) | 8.307692308 | 0.003947752 | 0.010856318 |
| 02-February_22 (n=6) | 06-June_22 (n=6) | 0.923076923 | 0.336668368 | 0.389826531 |
| 02-February_22 (n=6) | 07-July_22 (n=6) | 8.307692308 | 0.003947752 | 0.010856318 |
| 02-February_22 (n=6) | 08-August_22 (n=6) | 8.307692308 | 0.003947752 | 0.010856318 |
| 02-February_22 (n=6) | 09-September_22 (n=6) | 2.564102564 | 0.109314576 | 0.156842653 |
| 02-February_22 (n=6) | 10-October_22 (n=6) | 5.025641026 | 0.024974679 | 0.049949359 |
| 02-February_22 (n=6) | 11-November_22 (n=6) | 5.025641026 | 0.024974679 | 0.049949359 |
| 02-February_22 (n=6) | 13-January_23 (n=6) | 8.307692308 | 0.003947752 | 0.010856318 |
| 03-March_22 (n=6) | 04-April_22 (n=6) | 2.076923077 | 0.149541355 | 0.186221309 |
| 03-March_22 (n=6) | 05-May_22 (n=6) | 4.333333333 | 0.037372988 | 0.070474778 |
| 03-March_22 (n=6) | 06-June_22 (n=6) | 7.41025641 | 0.006485308 | 0.016462705 |
| 03-March_22 (n=6) | 07-July_22 (n=6) | 0.230769231 | 0.630954041 | 0.650671355 |
| 03-March_22 (n=6) | 08-August_22 (n=6) | 8.307692308 | 0.003947752 | 0.010856318 |
| 03-March_22 (n=6) | 09-September_22 (n=6) | 5.025641026 | 0.024974679 | 0.049949359 |
| 03-March_22 (n=6) | 10-October_22 (n=6) | 2.564102564 | 0.109314576 | 0.156842653 |
| 03-March_22 (n=6) | 11-November_22 (n=6) | 3.102564103 | 0.078169086 | 0.128978992 |
| 03-March_22 (n=6) | 13-January_23 (n=6) | 1.641025641 | 0.200184804 | 0.240221765 |
| 04-April_22 (n=6) | 05-May_22 (n=6) | 3.692307692 | 0.054663936 | 0.094942625 |
| 04-April_22 (n=6) | 06-June_22 (n=6) | 8.307692308 | 0.003947752 | 0.010856318 |
| 04-April_22 (n=6) | 07-July_22 (n=6) | 8.307692308 | 0.003947752 | 0.010856318 |
| 04-April_22 (n=6) | 08-August_22 (n=6) | 8.307692308 | 0.003947752 | 0.010856318 |
| 04-April_22 (n=6) | 09-September_22 (n=6) | 8.307692308 | 0.003947752 | 0.010856318 |
| 04-April_22 (n=6) | 10-October_22 (n=6) | 8.307692308 | 0.003947752 | 0.010856318 |
| 04-April_22 (n=6) | 11-November_22 (n=6) | 6.564102564 | 0.01040562 | 0.023681756 |
| 04-April_22 (n=6) | 13-January_23 (n=6) | 8.307692308 | 0.003947752 | 0.010856318 |
| 05-May_22 (n=6) | 06-June_22 (n=6) | 8.307692308 | 0.003947752 | 0.010856318 |
| 05-May_22 (n=6) | 07-July_22 (n=6) | 8.307692308 | 0.003947752 | 0.010856318 |
| 05-May_22 (n=6) | 08-August_22 (n=6) | 8.307692308 | 0.003947752 | 0.010856318 |
| 05-May_22 (n=6) | 09-September_22 (n=6) | 8.307692308 | 0.003947752 | 0.010856318 |
| 05-May_22 (n=6) | 10-October_22 (n=6) | 8.307692308 | 0.003947752 | 0.010856318 |
| 05-May_22 (n=6) | 11-November_22 (n=6) | 8.307692308 | 0.003947752 | 0.010856318 |
| **Group 1** | **Group 2** | **H** | **p-value** | **q-value** |
| 05-May_22 (n=6) | 13-January_23 (n=6) | 8.307692308 | 0.003947752 | 0.010856318 |
| 06-June_22 (n=6) | 07-July_22 (n=6) | 8.307692308 | 0.003947752 | 0.010856318 |
| 06-June_22 (n=6) | 08-August_22 (n=6) | 3.692307692 | 0.054663936 | 0.094942625 |
| 06-June_22 (n=6) | 09-September_22 (n=6) | 0.641025641 | 0.423339642 | 0.473566379 |
| 06-June_22 (n=6) | 10-October_22 (n=6) | 2.564102564 | 0.109314576 | 0.156842653 |

**Table S5.**

| **Parameter** | **Monthly Variation (KW)** | **Seasonal Variation (Mann-Whitney)** |
| --- | --- | --- |
| Water temperature | **0.0000** | **0.0000** |
| DO | 0.0668 | **0.0345** |
| Salinity | **0.0112** | 0.0575 |

**Table S6.**

| **Parameter** | **z-value** | **n₁** | **n₂** | **N total** | **r = z/√N** | **Interpretation** |
| --- | --- | --- | --- | --- | --- | --- |
| **Water Temperature** | -6.313 | 31 | 24 | 55 | -0.851 | Large effect |
| **DO** | -2.115 | 23 | 21 | 44 | -0.319 | Medium-low effect |

**Table S7.**

| **Parameter** | **Temperature** | **DO** | **Salinity** |
| --- | --- | --- | --- |
| **Temperature** | 1.000 | 0.172 | 0.240 |
| **DO** | 0.172 | 1.000 | -0.194 |
| **Salinity** | 0.240 | -0.194 | 1.000 |

**Table S8.**

| **Pair** | **n_a** | **n_b** | **Pseudo-F** | **R2** | **Permutations** | **p-value** | **p-value (BH)** | **p-value (Holm)** |
| --- | --- | --- | --- | --- | --- | --- | --- | --- |
| Mussels vs Biofilm | 72 | 72 | 49.6576738 | 0.259095672 | 99 | 0.01 | 0.01 | 0.03 |
| Mussels vs Seawater | 72 | 72 | 218.9250912 | 0.606566561 | 99 | 0.01 | 0.01 | 0.03 |
| Biofilm vs Seawater | 72 | 72 | 62.43410428 | 0.305399652 | 99 | 0.01 | 0.01 | 0.03 |

**Table S9.**

| **Protein name** | **Accession numbers (NCBI)^b^** | **Peptides^a^** | **Total**  **intensity^a^** | **Molecular**  **weight^b^** | **Mascot score^b^** | **Query^b^**  **coverage** |
| --- | --- | --- | --- | --- | --- | --- |
| Filamin-A | XP_052093719.1 | 178 | 11611830 | 284 722 | 4765 | 99% |
| Catchin protein | XP_063446251.1 | 107 | 3600562 | 227 984 | 1105 | 99% |
| LIM domain-containing protein | XP_052064529.1 | 42 | 1436134 | 130 422 | 1147 | 99% |
| PDZ domain-containing protein | XP_052070627.1 | 25 | 1218810 | 58 727 | 416 | 87% |
| Uncharacterized protein LOC110457451 isoform X2 | XP_052075383.1 | 26 | 932678 | 71 070 | 249 | 59% |
| Dihydropyrimidinase-like isoform X2 | XP_052065534.1 | 14 | 231360 | 63 558 | 1121 | 99% |
| Collagen alpha-1 | XP_052084727.1 | 3 | 73441 | 83 021 | 281 | 68% |
| Retrograde protein | XP_052092136.1 | 14 | 151025 | 66 292 | 729 | 99% |
| Kielin/chordin-like protein | XP_052693676.1 | 3 | 81992 | 41 960 | 252 | 91% |
| Protease inhibitor-like protein | XP_052071276.1 | 9 | 60848 | 160 303 | 1623 | 99% |
| Far upstream element-binding protein 3-like isoform X5 | XP_052091444.1 | 6 | 60024 | 73 977 | 659 | 83% |
| Calumenin-like isoform X2 | XP_052065479.1 | 3 | 55842 | 37 598 | 588 | 87% |
| Prickle-like protein 3 isoform X6 | XP_063403213.1 | 6 | 55854 | 67 935 | 575 | 93% |
| Byssal calumenin-like protein | XP_063411502.1 | 4 | 46807 | 36 813 | 471 | 92% |
| Uncharacterized protein LOC105318017 | XP_052091364.1 | 3 | 50024 | 15 376 | 92 | 92% |
| Mucin-16-like | XP_063421959.1 | 2 | 40248 | 89 2825 | 276 | 76% |
| Uncharacterized protein LOC105317552 | XP_063422588.1 | 3 | 31643 | 25 741 | 328 | 99% |
| LIM and SH3 domain protein F42H10.3-like isoform X15 | XP_052094657.1 | 1 | 28886 | 43 886 | 582 | 99% |
| Lysosomal alpha-mannosidase-like | XP_052059421.1 | 2 | 28851 | 126 421 | 1066 | 99% |
| Trichohyalin-like isoform X10 | XP_052066866.1 | 1 | 31537 | 83 988 | 679 | 76% |
| Fructose-1,6-bisphosphatase isozyme 2-like | XP_063433149.1 | 4 | 25711 | 36 644 | 655 | 99% |
| Proteasome subunit alpha type-5-like | XP_052079938.1 | 1 | 23837 | 26 454 | 486 | 99% |
| Apoptosis-inducing factor 3-like isoform X1 | XP_063396427.1 | 2 | 25422 | 10 1002 | 1120 | 99% |
| Calpain-A-like isoform X10 | XP_052058652.1 | 2 | 24552 | 90 884 | 1254 | 100% |
| Microtubule-associated protein 1B-like isoform X2 | XP_052102692.1 | 1 | 25099 | 167 677 | 1326 | 99% |
| Sarcoplasmic calcium-binding protein-like isoform X2 | XP_063421741.1 | 1 | 21859 | 20 602 | 161 | 74% |
| Neural cell adhesion molecule 2-like isoform X2 | XP_063440129.1 | 5 | 13030 | 126 565 | 994 | 99% |
| 17 beta-hydroxysteroid dehydrogenase | XP_063398333.1 | 2 | 15265 | 27 327 | 451 | 98% |
| Reticulon-1-A-like isoform X2 | XP_052106802.1 | 2 | 16385 | 40 384 | 538 | 98% |
| Endoribonuclease LACTB2-like | XP_052097194.1 | 2 | 18728 | 32 984 | 483 | 99% |
| Enoyl-CoA hydratase | XP_063446103.1 | 2 | 11764 | 32 191 | 515 | 97% |
| Eukaryotic translation initiation factor 4B-like | XP_052093520.1 | 2 | 16955 | 78 142 | 878 | 97% |
| Leucine-rich repeat-containing protein 40-like | XP_063430696.1 | 2 | 19066 | 52 758 | 662 | 94% |
| BTB/POZ domain-containing protein KCTD12-like | XP_063415524.1 | 2 | 7864 | 29 237 | 484 | 99% |
| Cilia- and flagella-associated protein 45-like isoform X1 | XP_052062857.1 | 2 | 9345 | 66 364 | 879 | 99% |
| Leucine-rich repeats and immunoglobulin-like domains protein | XP_063430697.1 | 1 | 11419 | 51 744 | 582 | 97% |
| Gelsolin-like protein | XP_052094229.1 | 1 | 9112 | 41 931 | 612 | 99% |
| SAP domain-containing ribonucleoprotein-like | XP_052095137.1 | 1 | 5956 | 26 606 | 277 | 88% |
| Elongation factor 1 alpha | XP_052097218.1 | 1 | 8175 | 50 299 | 890 | 99% |
| Mitochondrial H+ ATPase a subunit | XP_052101549.1 | 1 | 7821 | 59 379 | 713 | 100% |
| Nidogen-2-like | XP_063422474.1 | 1 | 9699 | 26 692 | 323 | 97% |
| Ubiquitin-associated protein 2-like isoform X9 | XP_052101254.1 | 1 | 5421 | 160 436 | 1989 | 99% |
| Mesenchyme-specific cell surface glycoprotein | XP_052101347.1 | 1 | 5715 | 62 779 | 684 | 95% |
| Phosphoglucomutase | XP_063415355.1 | 1 | 4075 | 68 249 | 1087 | 99% |
| Gamma-interferon-inducible lysosomal thiol reductase-like | XP_063407510.1 | 1 | 4225 | 29 767 | 362 | 93% |
| YLP motif-containing protein 1-like isoform X5 | XP_052069846.1 | 3 | 20564 | 108 544 | 1093 | 99% |
| Titin-like isoform X7 | XP_052064532.1 | 1 | 7894 | 122 256 | 265 | 91% |
| THO complex subunit 4-A-like | XP_052069589.1 | 1 | 8288 | 29 183 | 279 | 99% |
| CAT / catalase-like | XP_052087493.1 | 4 | 35456 | 57 886 | 1009 | 99% |
| Mn-superoxide dismutase | XP_063404002.1 | 1 | 1610 | 25 235 | 383 | 99% |
| Uncharacterized protein LOC127708635 isoform X2 | XP_052069628.1 | 3 | 29887 | 440 777 | 5436 | 99% |

**Table S10.**

| **Protein name** | **Accession number**  **(NCBI)^b^** | **Peptides^a^** | **Total intensity^a^** | **Molecular weight^b^** | **Mascot score^b^** | **Query coverage^b^** |
| --- | --- | --- | --- | --- | --- | --- |
| Uncharacterized protein LOC111134038 isoform X2 | XP_052101523.1 | 14 | 329321 | 147 388 | 2463 | 99% |
| LIM domain-containing protein | XP_052064529.1 | 11 | 229916 | 130 422 | 1147 | 99% |
| Aldehyde dehydrogenase | XP_052102174.1 | 5 | 78615 | 56 660 | 980 | 99% |
| Glutathione S-transferase A-like | XP_063407650.1 | 5 | 92070 | 26 338 | 373 | 99% |
| Uncharacterized protein LOC105341109 | XP_052083274.1 | 1 | 85491 | 44 250 | 604 | 99% |
| Uncharacterized protein LOC111111393 | XP_063400387.1 | 5 | 68748 | 36 140 | 588 | 99% |
| Peroxiredoxin-6-like | XP_063423659.1 | 4 | 74145 | 24 251 | 369 | 99% |
| Fatty acyl-CoA hydrolase precursor | XP_063444662.1 | 4 | 50442 | 65 616 | 613 | 99% |
| Glycogenin-1-like isoform X3 | XP_052104556.1 | 2 | 51149 | 41 110 | 621 | 92% |
| Protein disulfide-isomerase A6-like | XP_052086119.1 | 3 | 26942 | 47 105 | 478 | 99% |
| Transaldolase-like isoform X2 | XP_052086872.1 | 3 | 60643 | 36 575 | 593 | 99% |
| Uncharacterized protein LOC | XP_063446743.1 | 2 | 36817 | 29 307 | 329 | 99% |
| Tubulin beta chain | XP_052105089.1 | 1 | 24040 | 49 819 | 936 | 99% |
| xaa-Pro dipeptidase-like | XP_063441515.1 | 2 | 22655 | 55 470 | 926 | 99% |
| Proteasome subunit alpha type-7 | XP_052061881.1 | 2 | 15511 | 28 022 | 459 | 91% |
| Drebrin-like protein | XP_052099388.1 | 2 | 15745 | 62 428 | 560 | 63% |
| Uncharacterized protein LOC | XP_063424571.1 | 2 | 12878 | 285 267 | 3630 | 98% |
| Proteasome subunit alpha type-2-like | XP_052091406.1 | 2 | 18391 | 26 081 | 485 | 99% |
| Beta-arrestin-1-like isoform X1 | XP_063428875.1 | 2 | 16512 | 47 328 | 850 | 99% |
| Indolethylamine N-methyltransferase-like | XP_063426354.1 | 2 | 20352 | 29 232 | 293 | 99% |
| 4-hydroxyphenylpyruvate dioxygenase | XP_052073924.1 | 1 | 16096 | 43 482 | 716 | 96% |
| Laccase-2-like | XP_063430471.1 | 1 | 8481 | 79 929 | 893 | 97% |
| Uncharacterized protein LOC105339706 isoform X2 | XP_063411117.1 | 1 | 8428 | 101 162 | 100 | 63% |
| Vasodilator-stimulated phosphoprotein-like isoform X7 | XP_063427737.1 | 2 | 10735 | 47 126 | 265 | 39% |
| Uncharacterized protein LOC110450530 | XP_052099734.1 | 1 | 15913 | 32 905 | 147 | 82% |
| Microtubule-associated protein RP/EB family | XP_052063128.1 | 1 | 18709 | 30 737 | 503 | 99% |
| Proteasome subunit beta type-3-like | XP_052091747.1 | 2 | 8967 | 23 088 | 422 | 99% |
| Leucine-rich repeats and immunoglobulin-like domains protein | XP_063430697.1 | 1 | 16450 | 51 744 | 582 | 97% |
| Glucose-6-phosphate 1-dehydrogenase-like isoform X1 | XP_063447916.1 | 2 | 20194 | 62 962 | 993 | 99% |
| Uncharacterized protein LOC110460687 | XP_052062284.1 | 1 | 13780 | 36 039 | 506 | 99% |
| 1-deoxyxylulose-5-phosphate synthase YajO-like | XP_052096120.1 | 1 | 12428 | 37 188 | 593 | 95% |
| Trifunctional enzyme subunit alpha | XP_052058391.1 | 1 | 8639 | 83 018 | 1313 | 99% |
| Long-chain specific acyl-CoA dehydrogenase | XP_063424667.1 | 1 | 14201 | 48 467 | 785 | 96% |
| cAMP-dependent protein kinase catalytic subunit alpha isoform X1 | XP_052090805.1 | 2 | 11635 | 48 868 | 848 | 99% |
| Proteasome subunit beta type-2-like | XP_063432778.1 | 2 | 27371 | 22 522 | 383 | 87% |
| Sodium/potassium-transporting ATPase subunit alpha-like | XP_052062640.1 | 2 | 13432 | 114 139 | 1985 | 99% |
| Galactocerebrosidase-like isoform X1 | XP_063434432.1 | 1 | 13115 | 77 059 | 1095 | 99% |
| Coagulation protein factor 5 8 type domain-containing | XP_063405960.1 | 1 | 10647 | 27 578 | 132 | 54% |
| Leucine rich repeat only protein | XP_052058611.1 | 1 | 10216 | 50 673 | 575 | 91% |
| Ribosyldihydronicotinamide dehydrogenase | XP_052105464.1 | 1 | 7403 | 30 636 | 365 | 96% |
| Leukotriene A-4 hydrolase-like | XP_063422027.1 | 1 | 3392 | 72 730 | 993 | 99% |
| Annexin A4-like | XP_052059321.1 | 1 | 6293 | 61 970 | 612 | 99% |
| Hydroxyacyl-coenzyme A dehydrogenase | XP_052070005.1 | 1 | 6857 | 33 738 | 580 | 99% |
